# Ventilation-induced changes correlate to pulmonary vascular response and VEGF, VEGFR-1/2, and eNOS expression in the rat model of postnatal hypoxia

**DOI:** 10.1590/1414-431X20187169

**Published:** 2018-10-08

**Authors:** R.L. Figueira, F.L. Gonçalves, A.R. Prado, M.C. Ribeiro, K.M. Costa, O. Castro e Silva, L. Sbragia

**Affiliations:** 1 Laboratório de Cirurgia Fetal e Neonatal, Departamento de Cirurgia e Anatomia, Faculdade de Medicina de Ribeirão Preto, Universidade de São Paulo, Ribeirão Preto, SP, Brasil; 2 Laboratório de Transplante de Fígado, Departamento de Cirurgia e Anatomia, Faculdade de Medicina de Ribeirão Preto, Universidade de São Paulo, Ribeirão Preto, SP, Brasil

**Keywords:** Neonatal hypoxia, Mechanical ventilation, VEGF, VEGF receptors, eNOS enzyme

## Abstract

Neonatal asphyxia occurs due to reduction in oxygen supply to vital organs in the newborn. Rapid restoration of oxygen to the lungs after a long period of asphyxia can cause lung injury and decline of respiratory function, which result from the activity of molecules that induce vascular changes in the lung such as nitric oxide (NO) and vascular endothelial growth factors (VEGF). In this study, we evaluated the pulmonary and vascular morphometry of rats submitted to the model of neonatal asphyxia and mechanical ventilation, their expression of pulmonary VEGF, VEGF receptors (VEGFR-1/VEGFR-2), and endothelial NO synthase (eNOS). Neonate Sprague-Dawley rats (CEUA #043/2011) were divided into four groups (n=8 each): control (C), control submitted to ventilation (CV), hypoxia (H), and hypoxia submitted to ventilation (HV). The fetuses were harvested at 21.5 days of gestation. The morphometric variables measured were body weight (BW), total lung weight (TLW), left lung weight (LLW), and TLW/BW ratio. Pulmonary vascular measurements, VEGFR-1, VEGFR-2, VEGF, and eNOS immunohistochemistry were performed. The morphometric analysis showed decreased TLW and TLW/BW ratio in HV compared to C and H (P<0.005). Immunohistochemistry showed increased VEGFR-2/VEGF and decreased VEGFR-1 expression in H (P<0.05) and lower eNOS expression in H and HV. Median wall thickness was increased in H, and the expression of VEGFR-1, VEGFR-2, VEGF, and eNOS was altered, especially in neonates undergoing H and HV. These data suggested the occurrence of arteriolar wall changes mediated by NO and VEGF signaling in neonatal hypoxia.

## Introduction

Neonatal asphyxia is a pathological situation involving a deregulation of gas exchange resulting in hypoxemia, hypercapnia, and metabolic acidosis (1).

Despite important advances in perinatal care, asphyxia remains a severe condition, with high prevalence worldwide (2–4 per 1,000 live births) (2,3), leading to newborn mortality and morbidity (4). The statistics have predominantly focused on high-income countries, where research is most active. However, the greatest burden of the disease is in low- and middle-income countries (5).

In Brazil, asphyxia appears as the third or fourth cause of death in most states in 2015 and was the second leading cause of death in Maranhão (northeast region) (6). Although the mortality rate among newborns has decreased, the deficiencies of oxygen and trophic support very often affect various body organs and trigger long-lasting consequences that influence the quality of life (7).

There is a high prevalence of persistent pulmonary hypertension (PPHN) in asphyxiated neonates, and the long-term outcome of these infants depends on their underlying conditions and the therapeutic interventions received at birth (8). Rosenberg et al. found 6.4% hearing deficit and 24% respiratory morbidities in PPHN survivors (9). Although many studies have investigated the pathogenesis of asphyxia damage to the brain, research related to lung injury is scarce (10–14).

The molecular pathway leading to hypoxia-induced endothelial damage is probably related to the increase in the production of the hypoxia-inducible factor (HIF) (8), which facilitates adaptation to hypoxia (11 [Bibr B12]). One of the most important consequences of HIF hypersecretion in neonates affected by asphyxia is the increase in vascular endothelial growth factor (VEGF) expression (10).

VEGF acts by binding to two receptors, VEFGR-1 (Flt-1) and VEGFR-2 (Flk-1) (13). VEGFR-1 reduces cell proliferation and organizes capillary branching and networking, while VEGFR-2 is responsible for vascular cell proliferation and promotes vascular branching and maintenance of endothelial cells (14). VEGF can stimulate endothelial cells to produce nitric oxide (NO) by leading to the activation of endothelial NO synthase (eNOS) (15).

To date, the role of VEGF in the pathogenesis of hypoxia-induced pulmonary hypertension is not fully understood (16). Understanding the sequence of destructive events that are induced by hypoxia and what happens upon reoxygenation (ventilation) will allow the development of drugs that may inhibit these destructive molecular pathways.

Considering the importance of NO in the regulation of physiological pulmonary aspects, as well as the influence of hypoxia on the expression and activation of eNOS, VEGFR-1, VEGFR-2, and VEGF, the aim of the present study was to evaluate the morphometric changes in the lung and the pulmonary expression of these molecules in newborn rats submitted to neonatal hypoxia and mechanical ventilation. We hypothesized that isolated hypoxia and hypoxia plus ventilation have different effects on pulmonary vasculature, changing vascular response by vasodilatation and increased expression of VEGF and its receptors in the rat model of postnatal hypoxia.

## Material and Methods

### Ethical aspects

The study was approved by the Animal Experimentation Committee of the Faculdade de Medicina de Ribeirão Preto, Universidade de São Paulo (protocol #043/2011).

### Animals and pregnancy

Sprague-Dawley rats were mated overnight. The next day, a vaginal smear was obtained and mating was confirmed when a sperm smear was observed. This day was set as day zero of gestation (term=22 days). The animals were kept in cages with water and food *ad libitum*, under controlled illumination (12 h light/12 h dark), temperature (average 23°C), and relative humidity (average 55%).

### Model and experimental groups

A total of 8 pregnant rats were used to perform the study. We studied 4 groups with n=8 fetuses each: 1) control (C): healthy neonates without any type of intervention; 2) control submitted to ventilation (CV): healthy neonates submitted to mechanical ventilation; 3) hypoxia (H): neonates submitted to the hypoxia protocol; 4) hypoxia submitted to ventilation (HV): asphyxiated neonates submitted to mechanical ventilation. A total of thirty-two neonate rats were included in the study, all submitted to morphological analysis with 4 neonates per group subjected to histological analysis and 4 per group to immunohistochemistry analysis.

### Fetus harvesting

The fetuses were harvested at 21.5 days of gestation. On harvesting day, pregnant rats were anesthetized with 175 mg/kg ketamine (50 mg/mL, *im*) (Ketamina^®^, Pfizer do Brasil Ltda, Brazil) combined with 2.5 mg/kg xylazine (10 mg/mL) (Rompum^®^, Bayer Brasil, Brazil) and the fetuses were collected through a median laparotomy. If necessary, an extra dose (0.1 mL) of ketamine was administered intraperitoneally to the pregnant rat. Before any procedure, each fetus was weighed on an OHAUS model 360 precision scale (Denver Instruments, USA) and processed according to the specific procedures for each group.

**Figure 1:**
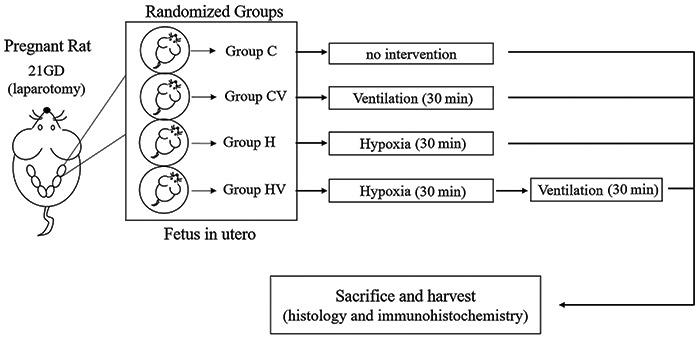
Study design. GD: gestation day; C: control; CV: control submitted to mechanical ventilation; H: hypoxia; HV: hypoxia submitted to mechanical ventilation.

Harvesting was performed through the following steps (Figure 1): 1) Two fetuses at a time were removed from the uterus and submitted to hypoxia for 30 min. 2) During this period, one fetus was removed from the uterus and harvested as C group (no manipulation). 3) After hypoxia, the two fetuses were separated, one was harvested as hypoxia group, and the other one was submitted to mechanical ventilation for 30 min. 4) At the end of the 30 min (ventilation), the fetus was harvest as HV group. To form the CV group, at step 1, one of the fetuses was submitted to mechanical ventilation for 30 min, while the other one was submitted to hypoxia.

### Induction of hypoxia

An anoxic acrylic chamber with a lid (30 × 20 × 12.5 cm) was used to induce hypoxia. The temperature was maintained at 38°C in a water bath. The chamber was filled with nitrogen (flow of 35 L/min) for 10 s, then flow was reduced to 5 L/min. (17) Groups H and HV were paired for a period of 30 min. After the induction of hypoxia, group H fetuses were euthanized by decapitation and placed on a cork table for harvesting, while group HV was submitted to mechanical ventilation (17).

### Mechanical ventilation

Mechanical ventilation was performed in fetuses of the CV and HV groups. The fetuses were placed in the supine position on a table with controlled temperature (average 38°C) and secured with adhesive tape. Using a surgical microscope with 12.5× magnification (D.F. Vasconcellos, Brazil), a tracheostomy was performed with a 24-G vascular Teflon catheter (BD Angiocath^TM^, Brazil) connected to a volume-cycled ventilator (MiniVent type 845, Harvard Apparatus^®^, Germany). The following parameters were used: 100% of oxygen, frequency of 80 breaths per minute, and 75 µL, which corresponds to moderate tidal volume (18). After confirming that chest movement was satisfactory, ventilation was applied for 30 min. At the end of this period, the fetuses were decapitated and placed on a cork table for dissection.

### Morphometric analysis

The following variables were measured: body weight (BW), total lung weight (TLW), and left lung weight (LLW). To remove the influence of BW on TLW we calculated the TLW/BW ratio.

### Histological and immunohistochemistry sample processing

The lungs were fixed in 10% formaldehyde and dehydrated in an increasing ethanol series (70, 80, 90, and 100%), cleared in xylene, and embedded in paraffin. Transverse 5-µm thick histological sections were obtained with a Leica Model RM 2145 microtome (Austria) and placed on pre-silanized histological slides.

### Vascular measurements

For morphometric analysis, the tissues were stained with Masson trichrome and the slides were mounted with Permount^®^ (Fisher Scientific). A Nikon Eclipse 80i photomicroscope with 200× magnification was used to obtain the images, which were then analyzed with the Image Pro Plus 6.0 software (Media Cybernetics, USA). The middle layers of preacinar arterioles measuring 30–60 μm in diameter were measured. The morphometric vascular parameters analyzed were external diameter (ED, μm) and internal diameter (ID, μm), which were used to calculate median wall thickness (MWT): MWT = (ED–ID)/ED (19).

### Immunohistochemistry for VEGF and VEGFR-1/R2

Tissues underwent antigen retrieval by boiling in an electric pan with 10 mM citrate buffer, pH 6.0, for 40 min. Endogenous peroxidase blockade was done with 3% hydrogen peroxide in phosphate-buffered saline (PBS). Nonspecific binding sites were blocked with 10% goat serum diluted in PBS for 1 h in a humid dark room. The samples were then incubated overnight at 4°C for 12 h with the primary antibodies diluted in 1.8% bovine serum albumin (BSA) (VEGF: mouse, 1:150, ab1316, Abcam, USA; VEGFR-1: rabbit, 1:50, ab2350, Abcam, USA; VEGFR-2: mouse, 1:25, sc6251, Santa Cruz Biotechnology, USA). After removal of the primary antibody, the appropriate secondary antibody (VEGFR-1: biotinylated goat anti-rabbit-sc-2040 antibody, Santa Cruz Biotechnology; VEGF and VEGFR-2: biotinylated goat anti-mouse antibody-sc-2005, Santa Cruz Biotechnology) diluted 1:200 in BSA was added. For the negative control, the primary antibody was omitted. The samples were then incubated for 30 min in streptavidin-HRP (Biolegend #405210, USA) diluted 1:200 in PBS. Next, they were developed with 3,3′-diaminobenzidine tetrahydrochloride (DAB, Sigma, USA) diluted 1:100 in hydrogen peroxide for 20 min. Finally, the samples were counterstained with Harris hematoxylin, washed in running water, dehydrated in an alcohol series and xylene, and covered with coverslips mounted with Permount^®^ (Fisher Scientific).

### Immunohistochemistry for eNOS

eNOS immunohistochemistry differed from VEGFR immunochemistry from the endogenous peroxidase blockade step onward. Blockade was carried out with 3% hydrogen peroxide in methanol and then with 10% goat serum in PBS for 30 min in a humid chamber. The sections were then incubated for 30 min with the ABC Kit (Vector Laboratories, USA) (20 μL of A and 20 μL of B) diluted in 5 mL of PBST (PBS + 0.1% polysorbate 20). The sections were then incubated overnight at 4°C for 12 h with the primary antibody anti-eNOS 1:100 (sc-654) (Santa Cruz Biotechnology, USA) diluted in 3% PBS/BSA. After removal of the primary antibody, the appropriate secondary antibody (biotinylated goat anti-rabbit antibody-sc-2004, Santa Cruz Biotechnology) diluted 1:200 in BSA was added. Next, the samples were developed with DAB. Finally, the slides were counterstained with hematoxylin, washed in running water, dehydrated in a sequential series of alcohol and xylol baths, and covered with coverslips mounted with Permount^®^ (Fisher Scientific, USA).

### Immunohistochemistry analysis

Forty images by group were analyzed. Images were captured using the Nikon Eclipse 80i photomicroscope with 400× magnification, then analyzed using Image J software (National Institutes of Health, USA) by the threshold tool to calculate the percentage of stained area focused on vascular areas.

### Statistical analysis

Morphometric and histometrical data were analyzed by ANOVA followed by the Tukey-Kramer post-test and are reported as means±SD. A P value <0.05 was considered significant. Analysis was performed using GraphPad Prism 5.0 software (GraphPad Software Inc., USA).

## Results

### Neonatal morphometry analysis

Mean BW, TLW, and TLW/BW ratio with their respective standard deviations are reported in Table 1. BW did not differ between control and intervention groups (hypoxia, ventilation, or both). The neonates from HV group had a significantly lower mean TLW and TLW/BW ratio compared to groups C and H (P<0.005 and P<0.05, respectively). The C group had the highest TLW/BW ratio and the HV group had the lowest TLW/BW ratio.

**Table 1:** Body weight (BW), total lung weight (TLW), and TLW / BW ratio (n=8 per group).

	BW (g)	TLW (g)	TLW / BW (%)
C	5.5290±0.5276	0.1727±0.0234**	0.0314±0.0043**
CV	5.4636±0.3189	0.1491±0.0249	0.0274±0.0052
H	5.6196±0.2123	0.1565±0.0222*	0.0279±0.0044*
HV	5.5263±0.5053	0.1214±0.0193	0.0220±0.0029

Data are reported as means±SD. C: control; CV: control submitted to mechanical ventilation; H: hypoxia; HV: hypoxia submitted to mechanical ventilation. *P<0.05, **P<0.005 compared to group HV (one-way ANOVA).

### Vascular measurements

**Figure 2:**
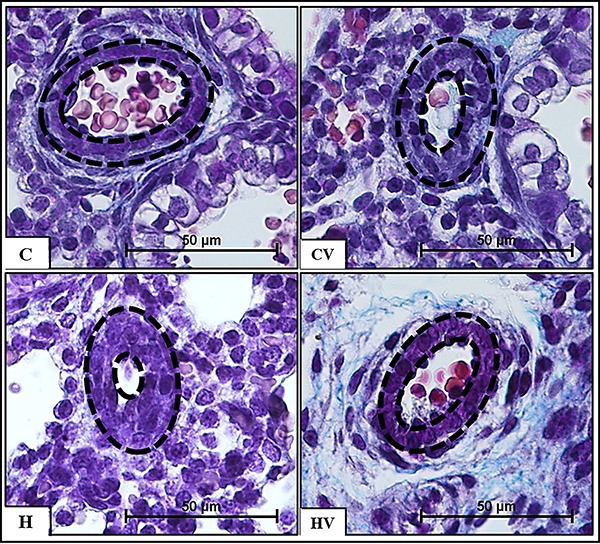
Photomicrographs of Masson's trichrome-stained arterioles. Dashed lines refer to the arteriole wall thickness. C: control; CV: control submitted to mechanical ventilation. H: hypoxia; HV: hypoxia submitted to mechanical ventilation. Magnification, 400×; bar, 50 μm.

Vascular morphometry analysis was performed by calculating the MWT of the preacinar arterioles measuring 30–60 μm in diameter (Figure 2). We analyzed 30 vessels per group in random fields from 4 neonates. MWT and its standard deviation obtained for each group are reported in Table 2. The MWT of the neonates that were submitted to the hypoxia protocol but that were not ventilated was significantly higher compared to the other 3 groups (P<0.05). MWT was significantly increased in the CV group compared to the C group and significantly reduced in the HV group compared to the H group (P<0.005).

**Table 2:** Mean wall thickness (MWT) of preacinar arterioles with diameter of 30–60 μm.

Groups	MWT (μm)
C	0.40667±0.09325*^a^ **^b^
CV	0.48125±0.10037*^bd^
H	0.55433±0.13841*^a^ **^cd^
HV	0.45113±0.11366**^b^

Data are reported as means±SD. C: control; CV: control submitted to mechanical ventilation. H: hypoxia; HV: hypoxia submitted to mechanical ventilation. *P<0.05, **P<0.005: ^a^compared to CV; ^b^compared to H; ^c^compared to HV; ^d^compared to C (one-way ANOVA).

### Immunohistochemical analysis of VEGFR-1, VEGFR-2, and eNOS

**Figure 3:**
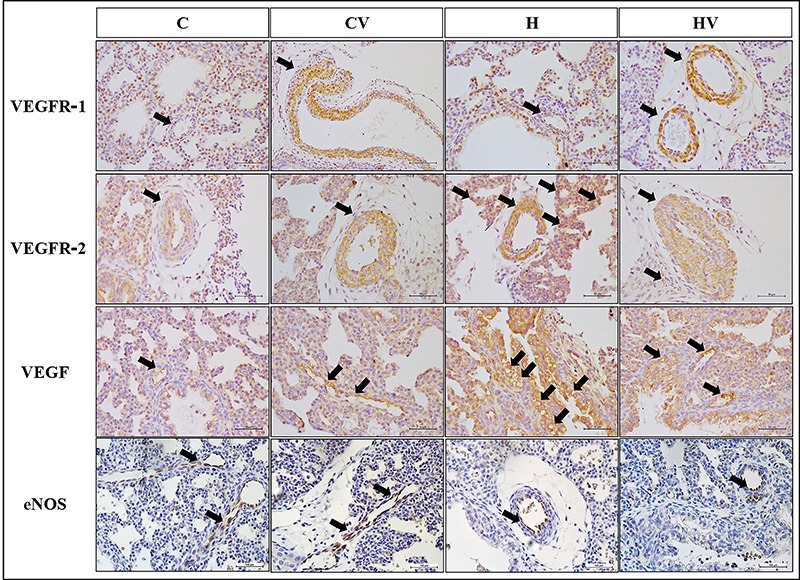
Immunostaining of vascular endothelial growth factors (VEGF)R-1, VEGFR-2, VEGF and endothelial NO synthase (eNOS) in the lung. C: control; CV: control submitted to mechanical ventilation. H: hypoxia. HV: hypoxia submitted to mechanical ventilation. Black arrows: local staining. Magnification, 200 ×; bar, 100 μm.

The immunohistochemical mean results and standard deviations are reported in Table 3. Negative controls showed no labeling. VEGFR-2 and VEGF expression presented similar results, both were higher in the H group compared to groups C, CV, and HV (P<0.05), and lower in the C group compared to the other groups (P<0.05). Group H exhibited a significantly lower VEGFR-1 expression compared to groups CV and HV (P<0.05), and a significantly higher expression compared to group C (P<0.05). Group C showed the lowest VEGFR-1 expression compared to the other groups (P<0.05). eNOS expression was higher in the CV group (P<0.001) and lower in groups H and HV (P<0.001) (Figure 3).

**Table 3:** Vascular endothelial growth factors (VEGF)R-1, VEGFR-2, VEGF, and endothelial NO synthase (eNOS) score by immunohistochemistry analysis.

	VEGFR-1 (μm^2^)	VEGFR-2 (μm^2^)	VEGF (μm^2^)	eNOS (μm^2^)
C	5488.5±916.8*^abc^	3979.8±1870.4**^abc^	2057.6±575.8**^abc^	4051.5±567.5**^bc^
CV	10478.0±1727.0*^bd^	8708.4±1757.2**^b^ *^d^	4119.2±2247.7**^bd^	4977.7±1841.6*^b^ **^c^
H	7031.0±759.7**^acd^	16615.0±5492.2**^acd^	11781.0±5511.6**^acd^	3014.7±308.3*^a^ **^cd^
HV	10355.8±1115.3*^bd^	7208.3±1215.3*^bd^	3436.0±2968.5*^bd^	2311.3±1055.6**^abd^

Data are reported as means±SD. C: control; CV: control submitted to mechanical ventilation; H: hypoxia; HV: hypoxia submitted to mechanical ventilation. *P<0.05, **P<0.005: ^a^compared to CV; ^b^compared to H; ^c^compared to HV; ^d^compared to C (one-way ANOVA).

## Discussion

The present study investigated and compared morphometrics, MWT, and pulmonary expression of VEGFR-1, VEGFR-2, VEGF, and eNOS in rat lungs under hypoxic conditions and after mechanical ventilation with 100% of oxygen.

There was no difference in BW between the groups. HV group presented lower TLW and TLW/BW ratio compared to C and H groups, suggesting the presence of a direct relationship between pulmonary weight and the hypoxic procedure followed by mechanical ventilation (20). Pulmonary ventilation injuries, already reported in the literature, are characterized by capillary leak, pulmonary endothelium or epithelium loss, escape of pulmonary fluid, tissue injury, inflammatory response, and edema. The major mechanical ventilation models are performed for long periods, resulting in edema and consequent gain of lung weight, making them inadequate to compare with the acute model performed by us. However, assuming the primary ventilatory effects, as the capillary and pulmonary fluid leaks, the acute ventilatory effects may promote the loss of weight, which was observed in HV group (21,22). In this study, we did not perform histological analysis of parenchyma, but focused on the vascular response and its biomarkers.

MWT was increased in the CV group compared to group C (P<0.05), suggesting an important effect of mechanical ventilation on pulmonary vascular changes. This is in agreement with Gonçalves et al. (23), who showed increased MWT of the pulmonary arterioles in ventilated groups compared to non-ventilated groups. Group H also showed increased MWT. Similarly, the studies of Sands et al. (24) observed this effect in asphyxiated newborn rats. Birth asphyxia has been shown to be associated with increased pulmonary vascular resistance due to the absence of the physiological elevation of oxygen tension at birth that represents an important factor in decreasing pulmonary vascular resistance after delivery (25). Consequently, the presence of pulmonary arterioles with the typical “fetal appearance” characterized by a thick muscular wall and a narrow lumen is observed in this scenario (10).

After 30 min of mechanical ventilation with 100% of oxygen in asphyxiated neonates (group HV), the vascular response (MWT) was similar to the CV group. The response on VEGFR-1, VEGFR-2, and VEGF expression was also similar in these two groups, which may suggest that short-term ventilation may be the inducible factor of these receptors. The expression of VEGFR-1 was higher and VEGFR-2 and VEGF expressions were lower in these groups (HV and CV). The explanation for this is the antagonistic response from VEGFR-1 and VEGFR-2.

It is known that VEGFR-2 is responsible for vascular growth, acting on the proliferation of vascular cells, promoting vascular branching and maintenance of endothelial cells (26–28). Otherwise, VEGFR-1 acts as a negative regulator of angiogenesis, reducing cell proliferation and organizing capillary branching and network formation. The performance of VEGFR-1 stems from its high affinity for VEGF, promoting sequestration thereof, and reducing the availability of VEGF for binding to VEGFR-2 (26–30).

The expressions of VEGFR-2 and VEGF were higher in group H compared to the other groups (P<0.05). This was also observed by Voelkel et al. (31) after hypoxic-ischemic events. These results are possibly associated with the modulation of VEGF, which is involved on the proliferation and migration process of endothelial cells. Our results on VEGFR-2 are also similar the findings from Tuder et al. (32), who reported that short-term exposure to hypoxia increased VEGFR-2 mRNA of isolated lungs. The increase of both VEGF and VEGFR-2 in H group indicates hypoxic pathway activation, in order to promote vascular growth and improve the oxygen saturation, since VEGF codification is modulated by the gene hypoxia-inducible factor (*HIF-2α*) (26,29,30,33).

Regarding eNOS, Jimenez et al. (34) showed that the mechanisms responsible for vascular changes are complex and different depending upon the oxygen tension, and under hypoxic conditions, flow-induced vasodilation is the result of not only nitric oxide, but of another unidentified oxygen-sensitive endogenous vasodilator in coronary arteries. It is possible that the same principle may apply to the pulmonary circulation and that might explain the study's findings regarding MWT decrease (vasodilation) with lower eNOS in the HV group.

Besides, eNOS enzymatic activity can be altered even if expression is unchanged. The NO signaling cascade is also affected under hypoxia. Thus, the lack of vasodilator response to VEGF may be due to multiple changes occurring at the receptor level down to the signaling cascade. Nadeau et al. (35) also suggested that a defect in receptor maturation may occur during hypoxia as hypoxia can affect the expression of glucose transporters, it could potentially affect the glycosylation of VEGFR-2.

These results indicated that VEGF played important roles in pulmonary microcirculation and that impaired eNOS-mediated relaxations may be a potential trigger of the onset of PPHN.

The study had some limitations, such as the lack of evaluation of parenchyma injury and lung volume weight, acute asphyxia (30 min), and short ventilation period (30 min). Also, the VEGF mRNA gene expression was not assessed.

Finally, in the rat model of neonatal hypoxia at term, hypoxia and mechanical ventilation altered vascular morphometry, proportionally to pulmonary expression of VEGF and its receptors (R1/R2), showing a significant alteration on MWT.

Understanding the molecular pathways that are altered in neonates submitted to hypoxia may outline promising pharmacological interventions that would reduce or even prevent the debilitating consequences of severe birth asphyxia, leading to better management and improved clinical outcome.
